# Exercise combined with pediatric Tuina and auricular acupressure for children with attention-deficit/hyperactivity disorder: a randomized controlled trial

**DOI:** 10.3389/fmed.2026.1880554

**Published:** 2026-07-17

**Authors:** Mingwei Xiong, Shuyun Jiang, Yonghao Sun, Xianzhu Cong, Nana Zhao, Fan Wu

**Affiliations:** 1 School of Clinical Medicine, Shandong Second Medical University, Weifang, China; 2 Affiliated Hospital of Shandong Second Medical University, Weifang, China; 3 School of Public Health, Shandong Second Medical University, Weifang, China

**Keywords:** acupressure, ear, attention deficit hyperactivity disorder, children, exercise, massage

## Abstract

**Objective:**

This study evaluated the clinical efficacy of three interventions for children with attention-deficit/hyperactivity disorder (ADHD): exercise alone, exercise combined with pediatric Tuina, and exercise combined with both pediatric Tuina and auricular acupressure.

**Methods:**

Participants were recruited from one senior kindergarten and two primary schools in Weifang City, Shandong Province, between December 2023 and June 2024. The Swanson, Nolan, and Pelham Rating Scale, 4th Revision (SNAP-IV) was used to assess symptoms. A total of 120 children aged 5–12 years diagnosed with ADHD according to the Diagnostic and Statistical Manual of Mental Disorders, Fifth Edition (DSM-5) were selected. Using SPSS 26.0 software, a randomization list was generated, and the children were randomly assigned to three groups (*n* = 40 each). Group A received exercise (skipping, jogging, shuttlecock kicking, basketball, badminton). Group B received exercise combined with pediatric Tuina. Group C received exercise, pediatric Tuina, and auricular acupressure. The SNAP-IV scale was re-administered at 1, 3, and 6 months post-treatment, and generalized estimating equations were used for statistical analysis. At the 6-months endpoint, the overall response rate and clinical control rate were compared among the groups.

**Results:**

Generalized estimating equations revealed no significant main effect of group (*P* > 0.05), but significant main effects of time (*P* < 0.001) and group-by-time interaction (*P* < 0.001) were observed. No significant differences in SNAP-IV scores were found among the three groups at baseline or after 1 month of treatment (*P* > 0.05). At 3 months, intergroup differences became significant (*P* < 0.05), with both Group B and Group C showing significantly lower scores than Group A (*P* < 0.05). At 6 months, GEE-based pairwise comparisons showed that Group C had a significantly lower estimated SNAP-IV score than Group A (adjusted *P* < 0.05), whereas the differences between Groups A and B and between Groups B and C were not statistically significant (adjusted *P* > 0.05). No group showed significant differences from itself. All three groups exhibited significant reductions in SNAP-IV scores at 1, 3, and 6 months post-treatment relative to baseline (all *P* < 0.05). At the 6-months endpoint, no significant difference was observed among the groups in overall response rate (*P* > 0.05), whereas the clinical control rate differed significantly across groups (*P* < 0.001). Specifically, Group C demonstrated a significantly higher clinical control rate than Group A (*P* < 0.001).

**Conclusion:**

All three interventions are effective in treating childhood ADHD. Compared with exercise alone, the exercise combined with pediatric Tuina and auricular acupressure intervention was associated with a greater reduction in SNAP-IV scores at 6 months and a higher clinical control rate; however, no statistically significant difference was observed between the exercise combined with pediatric Tuina intervention and exercise alone at 6 months.

## 1 Introduction

Attention-deficit/hyperactivity disorder (ADHD) is a common neurodevelopmental disorder characterized by developmentally inappropriate and persistent symptoms of inattention and/or hyperactivity-impulsivity that interfere with functioning or development (1). Epidemiological studies indicate that the prevalence of ADHD among school-aged children is approximately 5.3%, with no marked geographical variation. The disorder is consistently more common in males than in females. About 73.0% of affected children receive a formal diagnosis before age 18, and in nearly two-thirds of cases, symptoms persist into adulthood (2). Furthermore, adults with ADHD are at increased risk of developing antisocial personality disorders and other mental health conditions. Therefore, once a child is diagnosed, effective treatment should be provided to improve prognosis, enhance quality of life, and promote social stability.

Current treatment approaches for ADHD are generally classified into pharmacological and non-pharmacological interventions, with physical exercise considered a component of non-pharmacological or adjunctive interventions rather than an independent third category. First, pharmacological treatment is generally recommended as first-line therapy in school-aged children and adolescents according to major clinical guidelines, although the indication may vary by age group, symptom severity, and regional guideline recommendations, and it has been shown to reduce hyperactive behavior and improve cognitive function. However, sustained medication rarely leads to full recovery due to adverse effects, symptom rebound after discontinuation, and poor treatment adherence (3). Second, psychosocial interventions, including psychological therapy, parent training, and school-based interventions, are often combined with medication to reduce the risk of symptom relapse. These interventions, however, may be time-consuming, require substantial parental and professional involvement, and be difficult to sustain, particularly for families with limited time and resources (4). Physical exercise is increasingly recognized as a beneficial non-pharmacological adjunctive intervention for children with ADHD, rather than a standalone therapeutic category. Exercise may improve attention, inhibitory control, executive function, and emotional regulation through neurobiological mechanisms involving prefrontal and broader neural networks. A high-level meta-review has demonstrated that physical activity provides clinically relevant and neurobiological benefits across mental disorders and supports its use as part of multimodal treatment rather than as an ineffective intervention (5). While various other non-pharmacological modalities–such as cognitive training, neurofeedback, and complementary approaches–have been evaluated for pediatric ADHD, the strength and consistency of their evidence vary significantly (6). Furthermore, the feasibility and acceptability of these interventions often heavily depend on the child’s symptom severity and the specific intervention burden, meaning that compliance cannot be uniformly assumed without intervention-specific evidence.

Against this background, increasing attention has been directed toward non-pharmacological interventions for childhood ADHD, including exercise, pediatric Tuina, and auricular acupressure, and published clinical studies have provided preliminary evidence supporting the potential efficacy of pediatric Tuina and auricular acupressure in improving ADHD-related behavioral symptoms. Several randomized controlled trials have reported that pediatric Tuina may improve core ADHD symptoms such as hyperactivity and impulsivity in children (7, 8), while auricular acupressure has been shown to reduce behavioral problems and improve related functional outcomes in pediatric ADHD populations (9, 10).

Exercise has a comparatively established evidence base as an adjunctive intervention for children with ADHD, whereas emerging evidence from randomized and meta-analytic studies suggests that traditional non-pharmacological modalities such as pediatric Tuina and auricular acupressure may also provide beneficial effects on behavioral regulation and associated symptoms, although the certainty of evidence remains lower than that for exercise interventions.

Despite these differences, although individual studies and preliminary randomized controlled trials have suggested potential benefits of pediatric Tuina and auricular acupressure in ADHD populations (7–11), few controlled studies have directly compared exercise alone with exercise combined with pediatric Tuina or auricular acupressure, and it remains unclear whether the incremental burden of combined interventions results in proportionally greater symptom improvement. This uncertainty provides the rationale for conducting a three-arm trial to compare efficacy, feasibility, and acceptability across these modalities (12). Consequently, the comparative efficacy, feasibility, and appropriate application of these combined approaches remain uncertain.

## 2 Materials and methods

### 2.1 Research subjects

Participants were recruited from one senior kindergarten and two primary schools in Weifang City, China, between December 2023 and June 2024. Following the training of teaching staff and parents by pediatric specialists, preliminary screening for ADHD was conducted using the Swanson, Nolan, and Pelham Rating Scale, 4th Revision (SNAP-IV) (13). Following preliminary screening, eligibility was assessed by pediatric specialists. ADHD was confirmed in 120 children according to the diagnostic criteria of the Diagnostic and Statistical Manual of Mental Disorders, Fifth Edition (DSM-5) (14). The randomization table was generated using SPSS 26.0 software, and the participants were randomly allocated into three groups, with each group comprising 40 children. Participants were randomly allocated in a 1:1:1 ratio to three intervention groups. Group A received exercise alone; Group B received exercise combined with pediatric Tuina; and Group C received exercise combined with pediatric Tuina and auricular acupressure. The inclusion criteria were as follows: (1) a diagnosis of borderline or moderate ADHD according to the prespecified diagnostic criteria; (2) age between 5 and 12 years; (3) availability of at least one parent or legal guardian to assist with the home-based components of the intervention; and (4) written informed consent provided by a parent or legal guardian. The exclusion criteria were as follows: (1) severe organic disease, hematological disease, acute infectious disease, genetic disorder, or another psychiatric condition that could interfere with participation or outcome assessment; (2) use of medication or receipt of another ADHD-related intervention before enrolment; (3) concurrent participation in another clinical study; and (4) inability of the child or parent to understand or complete the study procedures at the time of enrolment.

Treatment retention and intervention adherence were evaluated separately. Retention was defined as completion of the SNAP-IV assessment at each scheduled follow-up, whereas adherence referred to completion of the prescribed intervention sessions. Follow-up assessments were coordinated through weekly digital video contacts, telephone reminders, and scheduled assessments at 1, 3, and 6 months. Parents recorded completed home-based sessions using a smartphone mini-program, and these records were reviewed weekly by the research team. All 120 randomized participants completed the scheduled outcome assessments and were therefore retained in the intention-to-treat analysis; no participant formally withdrew or was lost to outcome follow-up.

**FIGURE 1:**
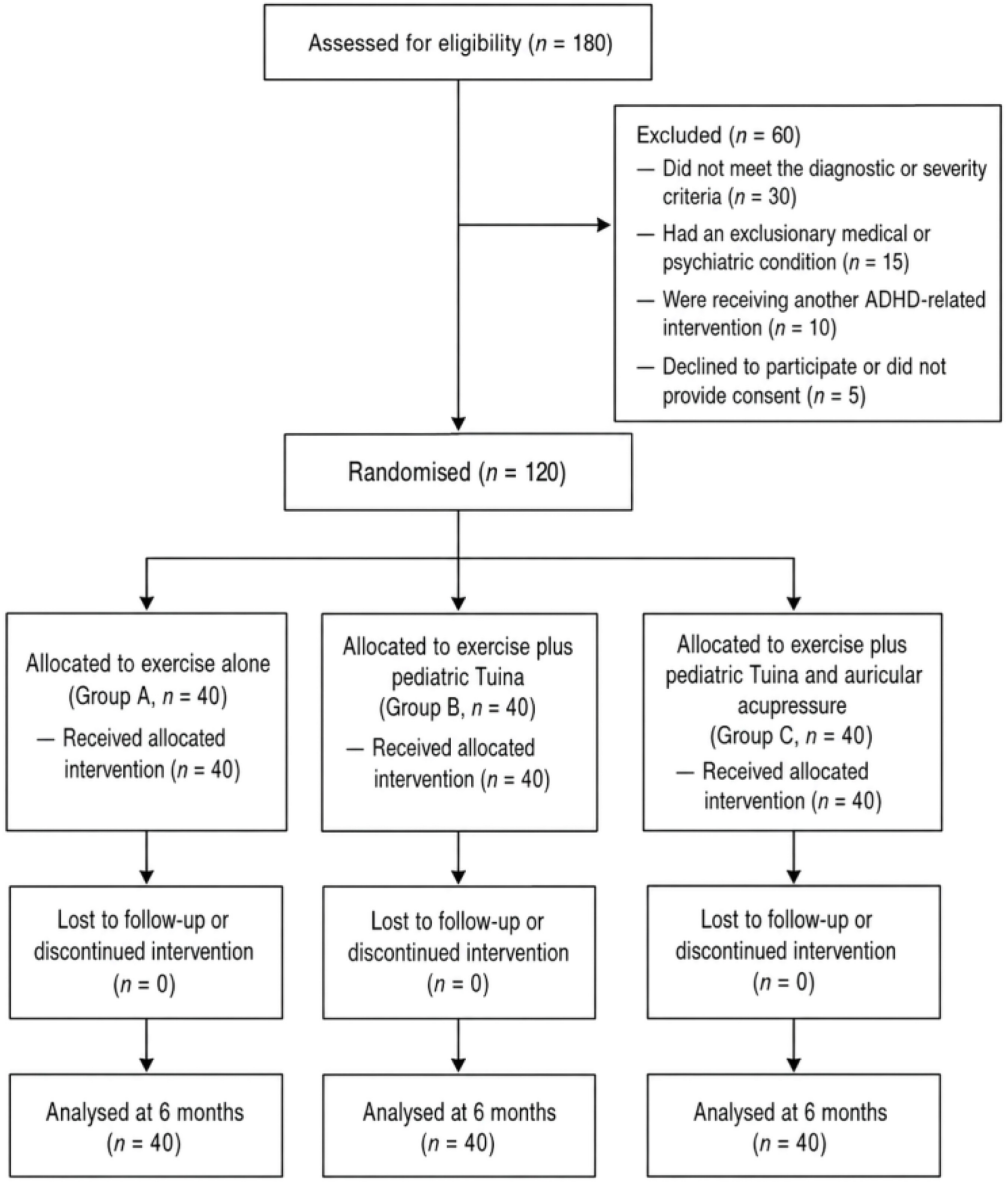
CONSORT flow diagram of participant screening, randomization, follow-up, and analysis.

Completion of outcome assessments did not imply complete adherence to the assigned interventions. Based on the electronic records, the illustrative mean proportions of prescribed sessions completed were 88.4% in Group A, 84.1% in Group B, and 80.6% in Group C. Protocol deviations mainly consisted of missed or shortened home sessions rather than treatment crossover or permanent discontinuation. Participants with missed intervention sessions remained in their originally assigned groups, and no participant was excluded from the primary analysis on the basis of adherence. Participant recruitment, eligibility assessment, randomization, follow-up, and analysis are summarized in the CONSORT flow diagram (Figure 1).

To control for potential confounding effects of concomitant treatments, information regarding pharmacological treatment (e.g., methylphenidate or other ADHD medications) and psychosocial interventions (e.g., behavioral therapy or parent training) was collected at baseline. No additional structured pharmacological or psychological interventions were initiated during the study period. Any ongoing treatments were required to remain stable for at least 4 weeks prior to enrollment and throughout the intervention period.

### 2.2 Intervention approach

#### 2.2.1 Exercise therapy

Participants engaged in moderate-intensity exercise 5 days per week. Activities were selected based on individual preferences and included skipping, jogging, shuttlecock kicking, basketball, and badminton; each session lasted 40 min (5).

#### 2.2.2 Massage therapy: pediatric Tuina

(1) Selection of acupoints: The following acupoints were selected: Baihui (GV20), Shenting (GV24), Sishencong (EX-HN1), Pishu (BL20), Banmen, Neibagua, Sihengwen, Xiaotianxin, Zhongwan (CV12), as well as the Bladder Meridian and Governor Vessel. (2) Operational procedure: For head and facial manipulation, the child was placed in a supine or seated position. The thumb tip or palm pad was used to knead Baihui and Shenting 150 times each, followed by kneading Sishencong 150 times. For upper limb manipulation, the following sequence was performed in a supine or seated position: Banmen was kneaded 200 times; Neibagua was performed 150 times; Sihengwen was pinched and kneaded 20 times; and Xiaotianxin was tapped 150 times. For chest and abdomen manipulation, Zhongwan (CV12) was kneaded 100 times, either in a supine or seated position. This was followed by tonification of the Spleen and Kidney meridians, performed 600 times. The procedure was then initiated in a prone position, using the heel of the palm as the pressure point. The Bladder Meridian and Governor Vessel pathways were kneaded along the back from the upper to the lower region for 3 min. All the above procedures were performed twice daily, with each treatment course repeated three to four times per week. (3) Implementation method: During the first 2 weeks, professional practitioners delivered the pediatric Tuina techniques in the hospital setting while simultaneously instructing the parents on the techniques. Subsequently, parents could choose to administer the therapy at home, or the treatment could continue to be performed in the hospital, depending on the child’s condition and parental preference.

#### 2.2.3 Auricular acupressure therapy

(1) Selection of acupoints: The following auricular points were selected: kidney, spleen, heart, liver, and Shenmen. (2) Procedure: After meticulous cleaning and disinfection of the patient’s auricle, the ear was stabilized with the left hand. Adhesive tape bearing Wangbuliu seeds was then picked up using mosquito forceps held in the right hand. The adhesive tape containing Wangbuliu seeds was positioned over each selected auricular acupoint. During the initial application, each acupoint was pressed for approximately 30 s, using sufficient pressure to produce a tolerable sensation of warmth, soreness, or distension without causing marked pain or skin injury. (3) Method of implementation: Parents were instructed to assist and supervise the child in pressing the taped auricular acupoints three times daily, preferably in the morning, afternoon, and evening. Each session lasted approximately 2 min, corresponding to a total daily stimulation time of approximately 6 min. During each session, the selected acupoints were pressed sequentially at a tolerable intensity sufficient to elicit mild warmth, soreness, or distension. Wangbuliu seeds were retained on one ear for 2 days and were then replaced on the contralateral ear. The two ears were treated alternately throughout the intervention period. Parents recorded each completed session in a treatment diary, which was reviewed during follow-up.

### 2.3 Research design

In this study, all children began treatment immediately after their initial diagnosis of ADHD. The intervention assigned to each group was predetermined and strictly allocated to the designated group. All children were reassessed using the SNAP-IV scale at 1, 3, and 6 months post-treatment. Teachers who completed the SNAP-IV assessments, as well as the assessors responsible for follow-up evaluations, were blinded to group allocation and intervention details. Completed assessment forms were coded using anonymous participant identifiers and analyzed by an independent coordinator. Data analysts had access only to the coded dataset and remained blinded until database lock.

### 2.4 Assessment of therapeutic effectiveness

The primary efficacy endpoint was assessed at 6 months based on the percentage reduction in SNAP-IV score, which was categorized as follows. A reduction of ≥80% with complete resolution of symptoms was classified as clinical control. A reduction of ≥50% with near-complete resolution of symptoms was classified as marked improvement. A reduction of ≥30% with symptomatic improvement relative to baseline was classified as improvement. A reduction of less than 30% with no significant clinical improvement was classified as ineffective. The percentage reduction in the SNAP-IV score was calculated as follows: [(baseline score − post-treatment score)/baseline score] × 100% (15). The overall response rate was calculated as: (number of cases with clinical control + marked improvement + improvement)/total number of cases × 100%.

### 2.5 Statistical methods

The normality of the data was assessed using SPSS version 26.0. For quantitative data following a normal distribution, descriptive statistics were presented as mean and standard deviation (e.g., for age), and group differences were analyzed using one-way analysis of variance. For quantitative data exhibiting non-normal distribution, descriptions were presented using median (M) with interquartile range (Q1: 25th percentile, Q3: 75th percentile). For ordinal categorical data, frequencies (n, %) were used for descriptive purposes, and group comparisons were conducted using Chi-square tests. The primary efficacy analysis followed the intention-to-treat principle and included all 120 randomized participants in the groups to which they were originally assigned. Missed or shortened intervention sessions were treated as protocol deviations and did not result in exclusion or reassignment. Because all participants completed the SNAP-IV assessments at baseline and at 1, 3, and 6 months, no outcome values required imputation. Generalized estimating equations (GEE) were used to estimate the population-averaged effects of group, time, and the group-by-time interaction. An independent working correlation structure and robust standard errors were specified. Estimated marginal means and corresponding 95% confidence intervals were obtained from the fitted GEE model. Bonferroni-adjusted contrasts were used for prespecified pairwise comparisons between groups at each time point and between time points within each group. Simple effects were evaluated within the GEE model framework using estimated marginal means (EMMEANS) with Bonferroni-adjusted contrasts for pairwise comparisons across time points and groups. The previously stated Friedman and Kruskal-Wallis tests were removed as they do not account for the correlation structure specified in the GEE model. In the present study, the statistical significance of *P* < 0.05 was considered, with all analyses conducted as two-tailed tests. The sample size was calculated based on detecting a medium effect size (Cohen’s *f* = 0.25) for repeated-measures ANOVA with three groups and four measurement points, using a significance level of α = 0.05, power (1−β) = 0.8, and anticipating a 10% dropout rate. This yielded a required sample size of 40 participants per group.

## 3 Results

### 3.1 Comparison of age and gender

No statistically significant differences were observed in age, sex, or baseline concomitant treatments (including pharmacological therapy and psychosocial interventions) among the three groups (all *P* > 0.05). At baseline, a small proportion of participants were receiving stable pharmacological treatment for ADHD (Group A: 12.5%, Group B: 10.0%, Group C: 12.5%), and some participants had previously received psychosocial behavioral training (Group A: 15.0%, Group B: 12.5%, Group C: 17.5%). No statistically significant differences were found among groups in either pharmacological or psychosocial treatment exposure (all *P* > 0.05), as illustrated in Table 1.

**TABLE 1:** Comparison of age and gender.

Basic information	Total (*n* = 120)	Group A (*n* = 40)	Group B (*n* = 40)	Group C (*n* = 40)	Statistic	*P*
Age, mean ± SD	8.82 ± 1.78	8.73 ± 1.75	8.92 ± 1.88	8.82 ± 1.73	*F* = 0.115	0.892
Gender, *n* (%)					χ^2^ = 0.137	0.934
Boy	103 (85.83)	34 (85.00)	34 (85.00)	35 (87.50)		
Girl	17 (14.17)	6 (15.00)	6 (15.00)	5 (12.50)		
Pharmacological treatment^a^, *n* (%)	13 (10.83)	5 (12.50)	4 (10.00)	5 (12.50)	χ^2^ = 0.12	>0.90
Psychosocial intervention^b^, *n* (%)	18 (15.00)	6 (15.00)	5 (12.50)	7 (17.50)	χ^2^ = 0.28	>0.80

*^a^*Pharmacological treatment refers to stable ADHD medication use (e.g., methylphenidate).

*^b^*Psychosocial intervention includes behavioral therapy or parent training programs prior to enrollment. F, analysis of variance; χ^2^, Chi-squared test; SD, standard deviation.

### 3.2 Comparison between time points before and after treatment

The GEE analysis showed that the main effect of group was not statistically significant (Wald χ^2^ = 3.679, *P* = 0.159), whereas the main effect of time (Wald χ^2^ = 1497.198, *P* < 0.001) and the group-by-time interaction (Wald χ^2^ = 109.380, *P* < 0.001) were statistically significant. Bonferroni-adjusted pairwise comparisons based on the GEE model showed no significant differences among the three groups at baseline or after 1 month of treatment (*P* > 0.05). Bonferroni-adjusted comparisons of GEE-estimated marginal means showed no statistically significant between-group differences at baseline or at 1 month (all adjusted *P* > 0.05). At 3 months, the estimated SNAP-IV scores were significantly lower in Group B and Group C than in Group A (both adjusted *P* < 0.05), whereas, based on the GEE-estimated marginal mean SNAP-IV scores at the 3-months follow-up, the difference between Groups B and C was not statistically significant (adjusted *P* > 0.05). At 6 months, Group C had a significantly lower estimated score than Group A (adjusted *P* < 0.05). The differences between Groups A and B and between Groups B and C were not statistically significant at 6 months (both adjusted *P* > 0.05).

Within-group comparisons based on the GEE model showed that SNAP-IV scores in all three groups were significantly lower at 1, 3, and 6 months than at baseline (all *P* < 0.05). Scores at 3 months were also significantly lower than those at 1 month, and scores at 6 months were significantly lower than those at 3 months in all three groups (all *P* < 0.05) (Table 2).

**TABLE 2:** Generalized estimating equations (GEE)-estimated marginal means of SNAP-IV scores by intervention group and time point.

Group	*n*	Baseline, EMM (95% CI)	1 month, EMM (95% CI)	3 months, EMM (95% CI)	6 months, EMM (95% CI)
Group A	40	35.4 (33.5–37.3)	34.5 (32.5–36.5)^a^	22.7 (20.4–25.0)^ab^	16.8 (14.0–19.6)^abc^
Group B	40	36.2 (34.2–38.2)	33.7 (31.6–35.8)^a^	17.8 (15.5–20.1)^ab^*	14.6 (11.8–17.4)^abc^
Group C	40	36.8 (34.6–39.0)	34.1 (31.9–36.3)^a^	15.8 (13.1–18.5)^ab^*	9.1 (6.8–11.4)^abc#^

Values are GEE-estimated marginal means with 95% confidence intervals. Superscripts indicate Bonferroni-adjusted within-group comparisons:

^a^compared with baseline;

^b^compared with 1 month;

^c^compared with 3 months (all adjusted *P* < 0.05).

*Significantly different from Group A at the same time point (adjusted *P* < 0.05).

^#^Significantly different from Group A at the 6-months time point (adjusted *P* < 0.05).

### 3.3 Comparison of overall response rate and clinical control rate

At the treatment endpoint, comparison of the overall response rates among the three groups revealed no statistically significant difference (*P* > 0.05). In contrast, a statistically significant difference was observed in clinical control rates across the three groups (*P* < 0.001), as shown in Table 3. Specifically, the difference in clinical control rates between Group A and Group C was statistically significant (*P* < 0.001), whereas the differences between Group A and Group B and between Group B and Group C were not statistically significant (both *P* > 0.05) (Table 4).

**TABLE 3:** Comparison of overall response rate and clinical control rate at treatment endpoint.

Treatment outcome	Group A	Group B	Group C	χ^2^	*P*
	(*n* = 40)	(*n* = 40)	(*n* = 40)		
Clinical control rate, *n* (%)				15.181	<0.001
Clinical control	7 (17.50%)	16 (40.00%)	24 (60.00%)		
Non-clinical control	33 (82.5%)	24 (60.00%)	16 (40.00%)		
Overall response rate, *n* (%)				4.418	0.126
Response	34 (85.00%)	37 (92.50%)	39 (97.50%)		
Non-response	6 (15.00%)	3 (7.50%)	1 (2.50%)		

χ^2^, Chi-squared test.

**TABLE 4:** Comparison of clinical control rates among groups at treatment endpoint.

Group	χ^2^	*P*	*P* ^#^
Group A vs. Group B	4.943	0.026	0.078
Group A vs. Group C	15.221	<0.001	<0.001
Group B vs. Group C	3.200	0.074	0.222

χ^2^, Chi-squared test, ^#^Bonferroni test.

## 4 Discussion

In the present study, the exercise-only intervention reduced ADHD symptoms over 6 months. Adding pediatric Tuina or auricular acupressure accelerated symptom improvement: at 3 months, both the exercise combined with pediatric Tuina intervention and the exercise combined with pediatric Tuina and auricular acupressure intervention showed significantly greater reductions in SNAP-IV scores than the exercise-only intervention. At 6 months, however, only the exercise combined with pediatric Tuina and auricular acupressure intervention remained significantly lower than the exercise-only intervention, whereas the difference between the exercise combined with pediatric Tuina intervention and the exercise-only intervention was no longer statistically significant.

Notably, no statistically significant differences were observed between the exercise combined with pediatric Tuina intervention and the exercise combined with pediatric Tuina and auricular acupressure intervention at any time point; however, the exercise combined with pediatric Tuina and auricular acupressure intervention group consistently demonstrated numerically better outcomes, particularly at the 6-months endpoint, suggesting a potential additive but non-linear effect of auricular acupressure that may not be fully captured within the current sample size. These temporal patterns suggest that while pediatric Tuina confers an early-onset advantage, its incremental benefit over sustained exercise alone may diminish over time, whereas the addition of auricular acupressure appears to provide a more durable adjunctive effect.

Clinical guidelines recommend behavioral interventions as first-line for young children with ADHD, with medication considered based on age, symptom severity, and functional impairment (16). Although pharmacotherapy is effective, adverse effects and poor long-term adherence limit its acceptability in some children. The World Federation of ADHD International Consensus Statement reviewed meta-analytic evidence on exercise for ADHD: one meta-analysis found a moderate symptom reduction that lost significance after adjustment for publication bias, whereas another reported no significant effects on core ADHD symptoms but improvements in anxiety and depression (17). Thus, exercise alone may offer supportive benefits but insufficient evidence supports it as a stand-alone treatment for core ADHD symptoms. In our study, exercise alone was associated with progressive SNAP-IV reductions over 6 months. The addition of pediatric Tuina (with or without auricular acupressure) produced earlier improvements (at 3 months), yet the advantage of exercise combined with pediatric Tuina over exercise alone disappeared by 6 months. This pattern suggests that sustained exercise may eventually yield comparable benefits, while the combination therapies accelerate the response. Because executive function and inhibitory control were not measured separately, no conclusions can be drawn about those specific domains.

From the perspective of TCM, ADHD symptoms are categorized under terms such as “restlessness” and “visceral agitation,” attributed to congenital deficiency, inadequate postnatal care, and yin-yang imbalance–often characterized as excess in heart and liver with deficiency in spleen and kidneys. Pediatric Tuina stimulates specific acupoints and meridians to harmonize qi and blood, balance yin and yang, and regulate visceral functions, thereby calming the mind and alleviating core symptoms. According to the *Compendium of Materia Medica*, “the brain is the residence of the original spirit”; when zang-fu dysfunction leads to qi and blood disharmony, the brain is inadequately nourished. Thus, treatment should address both the manifestation (brain) and the root (zang-fu dysfunction). In this study, Tuina was applied to head acupoints (Baihui, Shenting, Sishencong) along the Governor Vessel for immediate calming effects, followed by extra-meridian points (Banmen, Neibagua, etc.) to regulate zang-fu function, and finally back meridians to address the root. Chen et al. reported that parent-administered pediatric Tuina was feasible and beneficial, particularly for hyperactivity/impulsivity in preschool ADHD (7). Our results show that exercise combined with pediatric Tuina significantly reduced symptom scores at 3 months compared with exercise alone. However, by 6 months the difference was no longer significant, suggesting that the benefits of sustained exercise on executive function may gradually catch up. This multimodal approach has practical merits–good parent acceptability, easy home implementation, and no need for sophisticated resources–making it a promising non-pharmacological option.

Auricular acupressure is based on the *Huangdi Neijing* concept that “the ear is the convergence of meridians.” Stimulating auricular points (kidney, spleen, heart, liver, Shenmen) is believed to regulate zang-fu qi and blood, correct yin-yang imbalance, and calm the mind. Mahdavi et al. found that ear acupressure significantly reduced behavioral problems in children with ADHD (9), and Binesh et al. confirmed auricular therapy as an effective non-pharmacological intervention (10). In our triple therapy group (exercise combined with pediatric Tuina and auricular acupressure), sustained improvement was observed, but no statistically significant differences were found compared with the exercise combined with pediatric Tuina group in SNAP-IV scores or clinical control rates. This lack of additive effect may be due to overlapping therapeutic pathways between Tuina and auricular acupressure within the TCM framework. Therefore, adding auricular acupressure to exercise combined with pediatric Tuina did not provide measurable incremental benefit in this study, and its additional clinical value requires confirmation in larger, adequately powered trials.

Mechanistically, exercise may enhance systemic circulation and promote neurocognitive function, potentially improving attention and executive control. Pediatric Tuina and auricular acupressure may provide additional benefit through peripheral point stimulation. Recent qualitative evidence has shown that parent-administered pediatric Tuina can improve sleep quality, appetite, and overall well-being in early school-aged children with ADHD (8). Similarly, a randomized sham-controlled trial demonstrated that auriculotherapy significantly improved sleep quality in children with ADHD, with a significant time-intervention interaction effect (11). However, the precise neurobiological mechanisms underlying these clinical improvements remain unclear. It has been hypothesized that peripheral stimulation may influence local circulation and potentially affect central nervous system function through indirect hemodynamic or neuromodulatory pathways, but direct evidence of neurocognitive modulation specifically in children with ADHD is still lacking. Therefore, these proposed pathways should be interpreted as speculative and require further investigation using neuroimaging or neurophysiological approaches in pediatric ADHD populations.

Although the between-group differences between exercise combined with pediatric Tuina intervention group and the exercise combined with pediatric Tuina and auricular acupressure intervention group were not statistically significant, this finding does not necessarily indicate a lack of clinical value of auricular acupressure. First, the exercise combined with pediatric Tuina and auricular acupressure intervention group consistently demonstrated numerically better outcomes, particularly at the 6-months endpoint, suggesting a potential additive but non-linear effect of auricular acupressure that may not be fully captured within the current sample size. Second, auricular acupressure may provide incremental benefits in subdomains not fully captured by SNAP-IV, such as emotional regulation, sleep quality, and behavioral stability. Third, in pediatric behavioral interventions, small additive effects may accumulate over time and become more relevant in long-term management rather than short-term statistical comparisons. Therefore, auricular acupressure should be considered a low-cost, low-risk adjunct rather than an unnecessary burden.

Several limitations should be acknowledged. First, the sample size was relatively small. Second, the study included only children with borderline or moderate ADHD severity; however, ADHD-related comorbidities such as learning disabilities or emotional disorders were not specifically assessed or systematically excluded, although participants with severe psychiatric or organic conditions that could interfere with participation were excluded according to the study protocol. This may limit the generalizability of the findings. Third, the 6-months endpoint lacks long-term follow-up, so relapse risk and durability of effects remain unknown. Although all randomized participants completed the scheduled outcome assessments (100% retention for follow-up), this does not indicate perfect adherence to the prescribed interventions. Home-based delivery introduced variability in exercise intensity, Tuina technique, and acupressure frequency; some sessions were missed, shortened, or performed with differing intensity. These protocol deviations were retained in the intention-to-treat analysis and may have diluted or biased the treatment effect estimates. Future large-scale, rigorous randomized trials including children with severe ADHD or comorbidities are needed to confirm efficacy, optimize intervention strategies, and reduce reliance on medication.

## 5 Conclusion

At 3 months, both the exercise combined with pediatric Tuina (dual therapy) and the exercise combined with pediatric Tuina and auricular acupressure (triple therapy) produced significantly greater SNAP-IV reductions than exercise alone. At 6 months, however, only the triple therapy maintained a statistically significant advantage over exercise alone, whereas the dual therapy did not. The overall response rates did not differ significantly among the three groups, suggesting that all interventions were broadly effective in achieving symptomatic improvement. Notably, the clinical control rate was significantly higher in the triple therapy group than in the exercise–alone group, indicating that the triple combination was more likely to yield stringent remission. No statistically significant difference was found between the dual and triple therapies at any time point; nevertheless, the sustained benefit over exercise alone at 6 months was observed exclusively in the triple therapy group. These findings suggest that while adding pediatric Tuina confers an early improvement, the addition of auricular acupressure may contribute to maintaining longer-term symptom improvement trends, although its incremental benefit over Tuina combined with exercise alone did not reach statistical significance in the present sample. The present study did not assess socioeconomic factors or time-related treatment adherence; therefore, no recommendations can be made regarding intervention selection based on financial or time constraints.

## Data Availability

The raw data supporting the conclusions of this article will be made available by the authors, without undue reservation.
